# Effect of the Laser Cladding Parameters on Microstructure and Elevated Temperature Wear of FeCrNiTiZr Coatings

**DOI:** 10.3390/ma17184444

**Published:** 2024-09-10

**Authors:** Yali Gao, Sicheng Bai, Shan Jiang, Pengyong Lu, Dongdong Zhang, Meng Jie, Yu Liu

**Affiliations:** 1 Department of Mechanical Engineering, Northeast Electric Power University, No. 169 Changchun Road, Jilin 132012, China; dehuigyl@126.com (Y.G.); shshpa1998@163.com (S.B.); wasd1318143437@163.com (S.J.); lu971001@163.com (P.L.); yuliu@neepu.edu.cn (Y.L.); 2 School of Mechanical Electrical Engineering, Jilin Institute of Chemical Technology, Jilin 132022, China; jiemeng1980@126.com

**Keywords:** laser cladding, H13, high entropy alloy, microstructure, elevated temperature friction and wear

## Abstract

In order to prepare coating with good friction and wear resistance at elevated temperature on the surface of hot-working tool steel, by using a CO_2_ laser, FeCrNiTiZr high-entropy alloy coating with different laser scanning speeds (360, 480 and 600 mm/min, respectively) was successfully fabricated by using laser cladding technology on the surface of H13 steel in this paper. Phase constitutions, microhardness, microstructure, and wear characteristics of FeCrNiTiZr coatings under different laser scanning speeds were analyzed. It was determined that 480 mm/min was the optimal laser scanning speed. The results showed that the coating at the scanning speed of 480 mm/min consists of a BCC phase with significant lattice distortion and high dislocation density; the crystal structure is cellular crystal and dendrite crystal. The coating demonstrates the highest microhardness (842 HV_0.2_), which is 4.2 times that of the substrate (200 HV_0.2_). Its average friction coefficients at room temperature and 823 K are approximately one-seventh and one-third of the substrate’s, respectively, and its wear volume is reduced by about 98% and 81% under these conditions. Compared to the substrate, the coating underwent slight abrasive wear, adhesive wear, and oxidative wear at both room temperature and 823 K. In contrast, the substrate underwent severe abrasive wear, adhesive wear, oxidative wear, and even fatigue wear.

## 1. Introduction

H13 steel, recognized for its excellent heat resistance and impact toughness, is predominantly utilized in the manufacturing of die-casting molds [1,2,3]. Statistical evidence suggests that 70% of mold failures are attributable to wear, which not only causes a waste of resources but also escalates the costs of remanufacturing. However, since wear failures typically initiate at the surface, enhancing the mechanical properties of the mold surface has become a focal point of research [4,5]. Nowadays, the surface modification technologies mainly include thermal spraying [6], anodic oxidation [7], physical vapor deposition (PVD) [8,9], chemical vapor deposition (CVD) [10], and laser cladding [11,12,13,14,15,16,17,18,19]. Among them, laser cladding offers significant benefits including low dilution rates, rapid cooling, and robust metallurgical bonding [20,21]. Consequently, it is an effective method for repairing damaged areas and enhancing the surface wear resistance of H13 steel.

Laser cladding materials applied to H13 steel are primarily composed of single-component conventional alloys, including Ferric-based, Nickel-based, and Cobalt-based alloys [22,23,24,25,26]. These traditional alloys demonstrate limitations in reinforcing the resistance to wear and oxidation at elevated temperatures, leading to a gradual decline in research interest concerning these materials in recent years.

Metal–ceramic composite coatings which have high hardness and excellent elevated temperature wear resistance have been extensively researched. TiB, TiC, and WC are the main strengthening particles. Wang [27] discovered that directly adding TiC during the preparation of TiB_2_/Cu composite coatings significantly improved the material’s yield strength from 175 MPa to 422 MPa. Tran [28] used an in situ synthesis approach to produce TiB_2_/Cu composite coatings, finding that the even distribution of TiB2 particles enhanced the coating’s microhardness to 650 HV_0.5_. Zhao [29] investigated the effects of TiC content on TiC/Ni composite coatings, revealing that a TiC content of 30% yielded the highest hardness and best wear resistance. In another study, a blend of WC (WC-12Co) particles and Stellite-6 powder was utilized to create Stellite-6/WC composite coatings [30]. The incorporation of WC-12Co particles was found to reduce WC decomposition, significantly improving the coatings’ wear resistance.

Although the coating based on ceramic particles offers higher hardness to the surface of the cladding zone of the substrate, the large thermal expansion coefficient of ceramic particles, combined with their low impact resistance and poor post-processing capabilities, make metal–ceramic composite coatings unsuitable for service requirements of hot-working tool steel. Therefore, it is essential to search for a coating material that can enhance surface hardness, improve elevated temperature wear resistance, and facilitate subsequent processing.

High-entropy alloy (HEA) possesses numerous advantages, such as enhanced strength, hardness, thermal stability, and resistance to corrosion, which significantly enhance the overall performance of hot-working tool steel. However, current research on laser cladding HEA onto H13 steel primarily concentrates on microstructure analysis and room temperature friction and wear. Shu [31] explored how the amorphous content affects the performance of CoCrBFeNiSi alloy, showing that a higher amorphous content improved wear and corrosion resistance. Moreover, research [32] indicated that incorporating niobium into the Al_0.5_CoCrFeNi alloy produced an oversaturated structure, which led to finer coating grains and a notable improvement in hardness and wear resistance. Shi [33] used laser cladding to develop in situ NiCoCrMnFe high-entropy alloy coatings on H13 steel. The findings demonstrated that the NiCoCrMnFe coatings had a microhardness exceeding 500 HV, which is more than 2.5 times that of the base material. Compared with the substrate, the average width of wear scars on the coating decreased by 0.23 mm, and the wear rate of the coating reduced by 63.2% under the same friction conditions.

Existing research about the elevated temperature friction and wear performance of coatings is lacking, particularly in comparative analyses of wear resistance between coatings and H13 steel at varying experimental temperatures. Consequently, a new FeCrNiTiZr high-entropy alloy which has high hardness and good elevated temperature wear resistance was designed by referring to the classical FeCoCrNiAl high-entropy alloy system and fabricated on H13 steel. Its microstructural evolution and hardness were systematically studied; the elevated temperature wear mechanisms at varying experimental temperatures were especially investigated. The results show that this coating can be used as an excellent repair material for H13 hot-working tool steel.

## 2. Materials and Methods

### 2.1. Substrate Material

H13 steel was chosen as the substrate material (purchased from Kaiyiyou Metal Products Co., Ltd., Kunshan, China), and the dimension of substrate was 50 mm × 30 mm × 10 mm. The chemical composition of H13 is presented in Table 1. Initially, the surface of the substrate was milled and subsequently polished with sandpaper to eliminate impurities.

### 2.2. Coating Material

To meet the performance requirements of hot-working tool steel, a BCC-based high-entropy alloy coating was designed, which has high hardness and good elevated temperature wear performance. In this system, FeCoCrNiAl has been extensively studied, with Ni and Cr contributing to improved plasticity and performance at high temperatures. Moreover, Ni enhances the coating’s wettability with the substrate, reducing its brittleness. Fe is one of the most common metallic materials; it not only forms Fe-Cr solid solutions with Cr, which has exceptional properties, but also improves the compatibility between the coating and the substrate. Ti and Zr contribute to increased strength due to their larger atomic radii, which induce lattice distortion effects. Therefore, Ti and Zr were selected to replace Al, which reduces the coating hardness, and Co, which is prone to sintering. In summary, FeCrNiTiZr was designed as the coating material.

**Figure 1:**
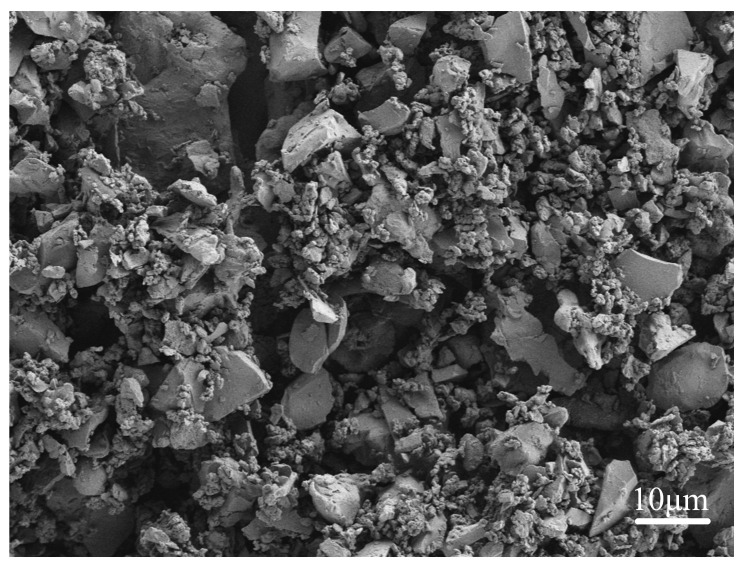
The morphology of FeCrNiTiZr powder after mixing.

The metal powders utilized in this research were procured from Xingtai Xinnai Metal Materials Co., Ltd. (Xingtai, China) The particle size of these powders is 300^#^, and purity levels are between 99.0% and 99.5%. According to Table 2, the powders were measured, then fully mixed in a ball mill (Honghong Instrument and Equipment Co., Ltd., Qidong, China) for two hours, followed by drying in a vacuum oven (Hebi Tianguan Instrumentation Co., Ltd., Hebi, China) for two hours. The microscopic morphology of the mixed powder is illustrated in Figure 1. A 0.7 mm thick layer of powder was presented to H13 steel using a mold with dimensions of 50 mm × 30 mm × 0.7 mm before laser cladding.

### 2.3. Laser Cladding Experiment Methods

**Figure 2:**
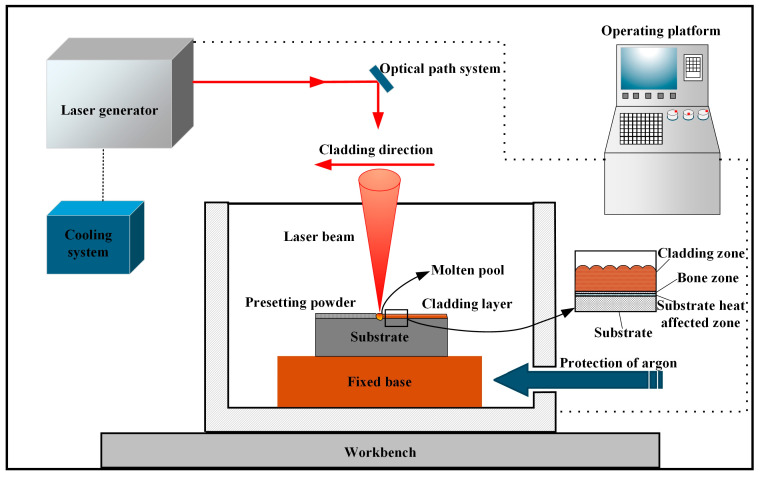
Schematic diagram of laser cladding process.

**Figure 3:**
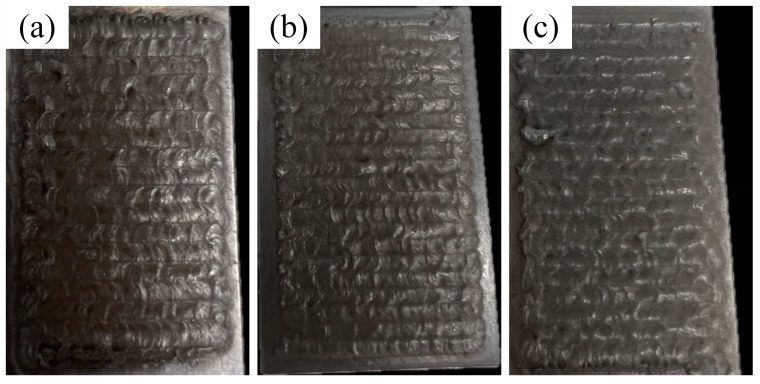
Cladding morphology under different scanning speeds: (**a**) 360 mm/min; (**b**) 480 mm/min; (**c**) 600 mm/min.

A schematic of the laser cladding process is depicted in Figure 2. The cladding experiments were conducted using a DL-2000 crossflow CO_2_ laser (Shenyang Continental Laser Complete Equipment Co., Ltd., Shenyang, China). The process parameters were determined as in Table 3. As depicted in Figure 3, the coatings at different scanning speeds have good morphology and show fish scales, and no cracks are observed.

### 2.4. Microstructure and Performance Analysis Methods

TD-3500 (Tongda Technology Co., Ltd., Dandong, China) X-ray diffractometer was used for phase analysis. The sample was cut to 10 mm × 10 mm × 10 mm before the experiment. The apparatus provided a Ni-filtered Cu Kα source. The experimental parameters are as follows: operating voltage is 40 kV, current is 30 mA, the data acquisition range is 20°~100°, the step size is 0.034, and the step time is 0.05 s.

A Zeiss Sigma 300 SEM (Zeiss, Oberkochen, Germany) was employed to capture the microstructure characteristics and the morphology of the worn surfaces. The elemental distribution of coatings was analyzed using an X-MAX50 energy dispersive spectrometer (EDS, Oxford, UK). The aqua regia (HNO_3_:HCl = 1:3) was selected as the corrosion solution (Chengdu Cologne Chemical Co., Ltd., Chengdu, China); the etching time was 60 s.

The HXD-1000TMC/LCD Vickers was used to measure the microhardness of coatings (Wuxi Metes Precision Technology Co., Ltd., Wuxi, China). The experiment with a load of 200 gf was applied for 15 s. Additionally, measurements were taken three times at 0.05 mm intervals along the depth of the sample.

The elevated temperature friction and wear tests were conducted using an MGW-02 wear tester (Jinan Yihua Tribology Testing Technology Co., Ltd., Jinan, China) at experimental temperatures of 298 K and 823 K, respectively. The test parameters included a load of 20 N, a frequency of 10 Hz, a sliding time of 20 min, and a reciprocal sliding distance of 3 mm. The grinding ball had a radius of 3.5 mm. The wear volume was calculated using the formulas provided in Equations (1) and (2).

(1) $$V=(\frac{1}{2}\theta R^{2}-\frac{1}{2}\sin\theta_{1}R^{2})\cdot L$$

(2) $$\theta_{1}=\arcsin\frac{L_{1}}{2R}$$

The parameters in the above formula are as follows: *θ* is arc degree; *R* is the radius of grinding ball; *θ*_1_ is the friction angle; *V* is the wear volume; *L* is the friction distance; *L*_1_ is the wear scar width.

## 3. Results

### 3.1. Phase Analysis

**Figure 4:**
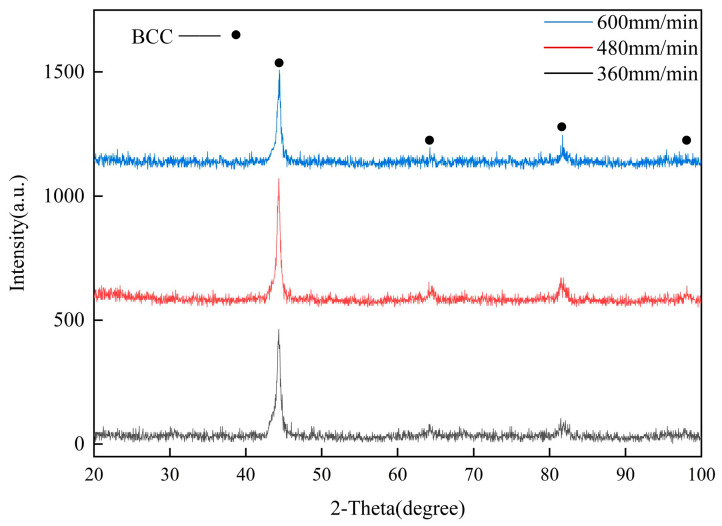
The XRD results of coatings.

The coatings’ XRD results are presented in Figure 4. Due to the unique high-entropy effect of high-entropy alloy, the mixing entropy of liquid alloy exceeds the critical formation entropy of intermetallic compounds, which prevents their formation [34]. Furthermore, laser cladding technology has the characteristic of rapid cooling rate, which suppresses the precipitation of intermetallic compounds. As a result, the analysis shows that the phases of the three coatings consist solely of a single BCC (Fe-Cr) phase, with no intermetallic compounds detected.

According to Bragg equation Formulas (3) and (4), the lattice constants (a) of the Fe-Cr (2θ = 43.966°) are calculated and displayed in Table 4. As exhibited, the lattice constants of Fe-Cr phases in coatings are higher than that of the normal lattice constant of Fe-Cr (2.876 Å) phase [35], which demonstrates that Fe-Cr phases produce lattice distortion in laser cladding.

(3) 2dsinθ=nλ

(4) $$d=\frac{\alpha }{\sqrt{h^{2}+k^{2}+l^{2}}}$$

where *n* is the diffraction series (*n* = 1); *d* is the crystal face distance; *λ* is the wavelength of X-ray (*λ* = 1.54056 Å), *α* is the actual lattice constant, *θ* is the diffraction angle.

For cubic crystals, the formula for calculating the lattice distortion (*ε*) is as follows [36]:

(5) $$\varepsilon =\frac{\left|\alpha -\alpha_{0}\right|}{\alpha_{0}}$$

In the above formula, *α_0_* represents the theoretical lattice constant. By calculation, the results of Fe-Cr phases are demonstrated in Table 4. The FeCoNiTiCr coatings in this study are derived from the most common HEA system (FeCoCrNiAl), and the lattice distortion of coatings are higher than that of FeCoCrNiAl HEA (7.0 × 10^−4^) [37].

This phenomenon is mainly because of the addition of Ti and Zr elements, which possess larger atomic radius. Additionally, the rapid solidification inherent to the laser cladding process causes the lattice distortion.

Based on the Gaussian distribution method and X-ray diffraction result [38], the dislocation density of coatings is determined. Dislocation density (*ρ*) is calculated as follows:

(6) $${\left(\frac{\beta \cos\theta }{\lambda }\right)}^{2}=\frac{1}{D^{2}}+16\xi^{2}{\left(\frac{\sin\theta }{\lambda }\right)}^{2}$$

where *θ* is the diffraction angle; *D* is the grain size; *β* is the half-height width; and *ξ* is the microscopic strain of the coating. *β* and *θ* are obtained by the measure results in XRD and as shown in Table 5. By graphing square numbers, the slope (16*ξ*^2^) and intercept (1/*D*^2^) of the line were derived, which facilitated the calculation of *ξ* and *ρ*(1/*D*^2^).

According to Formula (6), upon calculation, *ξ* and *ρ* of the Fe-Cr phase are shown in Table 4. It is evident that the dislocation density in the coating reached that of the work-hardened alloy, measuring between 10^11^ and 10^12^ cm^−2^. This is attributed to the influence of the laser beam, which interacted with the surface of substrate briefly, but it generated pressures up to 10^5^ atmospheres. This intense pressure induced significant plastic deformation and led to the production of high-density dislocations. Additionally, when the scanning speed reached 480 mm/min, the microstrain and dislocation density in coatings were maximum, indicating that the coating achieved its highest strength at this scanning speed.

### 3.2. Microstructure Analysis

**Figure 5:**
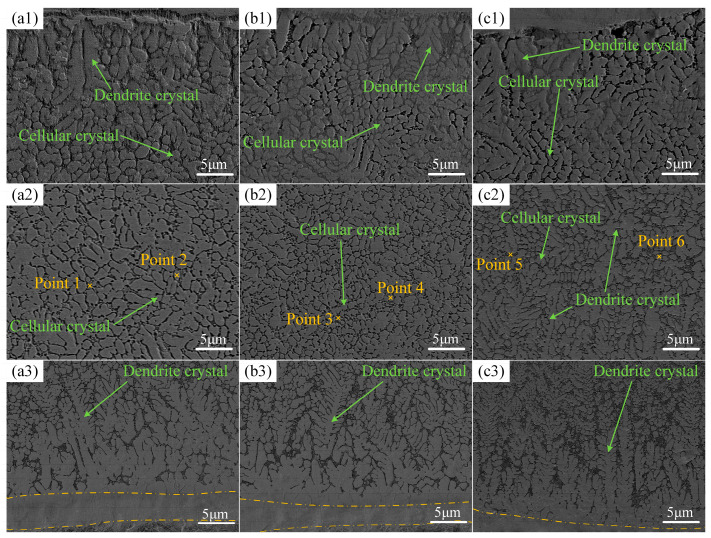
Microstructure of surface, middle parts, and bonding zone of each coating at different scanning speeds: (**a1**–**a3**) 360 mm/min; (**b1**–**b3**) 480 mm/min; (**c1**–**c3**) 600 mm/min.

The characteristics of the solidification microstructure are determined by the temperature gradient (G) and the solidification rate (R) [39]. Due to the heat dissipation by the substrate, G/R of the bottom part was higher than in other areas of the melt pool, impelling the production of planar crystals as indicated by the dashed regions in Figure 5(a3,b3,c3). The presence of planar crystals provided nucleation sites for dendrite crystal growth, which occurred along the temperature gradient’s direction. With the solid–liquid interface moving to the surface of the coating, the G/R ratio gradually decreased, resulting in a transition to cellular crystals, as shown in Figure 5(a2,b2,c2).

During the laser processing, a thin layer of Zirconium Oxide (ZrO2) formed on the coating surface, which also provided nucleation sites for dendrite crystals growth. Due to the enhanced cooling effect near the surface, the dendrite crystals formed in these areas were notably shorter, as illustrated in Figure 5(a1,b1,c1).

Furthermore, an analysis of Figure 5 reveals that the grain size is inversely proportional to the scanning speed. This is because the increase in scanning speed caused the heat accumulated by the coating to decrease, which also means that the cooling rate became faster, resulting in insufficient grain growth time. The changes in cellular crystals within the middle section of the coating were particularly pronounced. These uniformly distributed and fine cellular crystals led to an increase in strength of the coatings. However, as the scanning speed reached 600 mm/min, the excessive thermal gradient in the coating favored the formation of dendrite crystals over cellular crystals, reducing the proportion of cellular crystals, as shown in Figure 5(c2).

The point scanning results of coatings at different scanning speeds are shown in Table 6. In Figure 5(a2,b2,c2), Points 1, 3, and 5 represent grains, while Points 2, 4, and 6 represent grain boundaries. The analysis indicates that the elemental distribution within the grains of coatings is consistent at different scanning speeds, and similarly, the elemental distribution at the grain boundaries remains consistent. The high content of Fe was mainly due to the dilution effect, where the melting of the substrate during heating led to the diffusion of a significant amount of Fe into the coating, resulting in a noticeable increase in Fe content. In contrast, the Ti content significantly decreased, primarily because Ti has low corrosion resistance in media that generate hydrogen, such as aqua regia, leading to partial corrosion of Ti during the etching process with aqua regia. Comparison of the elements at grain boundaries and within grains reveals that Ti and Zr show significant segregation at the grain boundaries. This is because their larger atomic radii result in a relatively low solubility, but this segregation also restricts grain growth, thereby contributing to fine grain strengthening.

### 3.3. Microhardness Analysis

**Figure 6:**
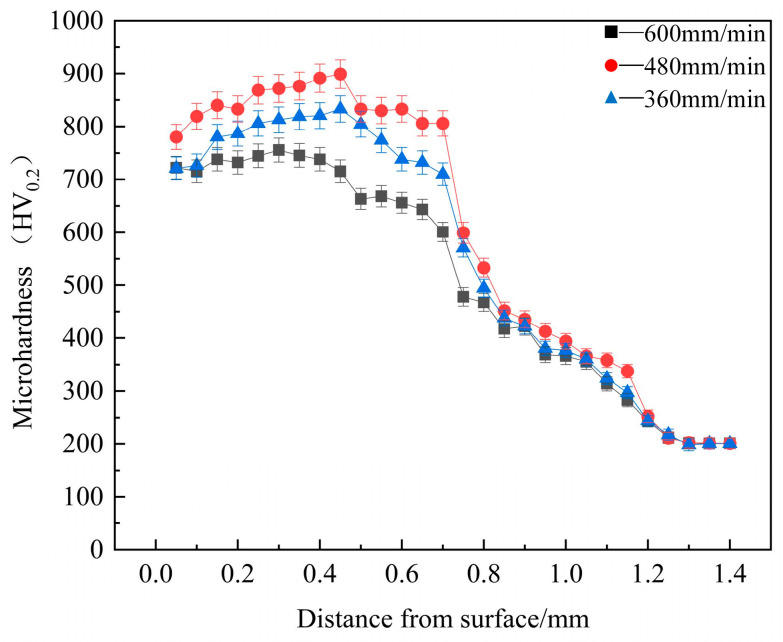
Cross-sectional microhardness of coatings.

The distribution of microhardness across the cross-sections of coatings at various scanning speeds is depicted in Figure 6. It is evident from the figure that the average hardness of the coatings was significantly higher than that of the substrate, surpassing it by 3.5 to 4.2 times. This enhancement is primarily due to the following reasons: Firstly, the FeCrNiTiZr coating comprised five elements with varying atomic radii, particularly the larger radii of Ti and Zr, which induced substantial lattice distortions and contributed to solid solution strengthening. Secondly, the swift rates of melting and cooling typical of laser cladding inhibited the growth of grain, thereby enhanced strength and hardness of coatings. Finally, the high dislocation density within the coating led to the accumulation of dislocations, which significantly impeded the initiation of slip systems. To sum up, the effects of solid solution, dislocation, and fine grain strengthening synergistically conferred increased hardness on the coatings.

At a scanning speed of 480 mm/min, the coating exhibited the highest average hardness (842 HV_0.2_), which was 4.2 times greater than that of the substrate (200 HV_0.2_). In addition, compared with the FeCoCrNiMnAl coating (512 HV_0.5_) [40] derived from the same system (FeCoCrNiAl), the microhardness increased by 64.5%. The coatings possessed elevated hardness due to the substantial accumulation of dislocations during the solidification process. Moreover, as demonstrated by the calculations in Table 4, the coating at this scanning speed had the highest dislocation density. This is further supported by SEM images of the microstructure. With the increase in scanning speed, the grains became finer, but the proportion of dendrite crystals increased when the scanning speed reached 600 mm/min, which limited the performance of the coating. This reveal that under such conditions, the coating’s grains are finer and more compact.

### 3.4. Wear Resistance Analysis

**Figure 7:**
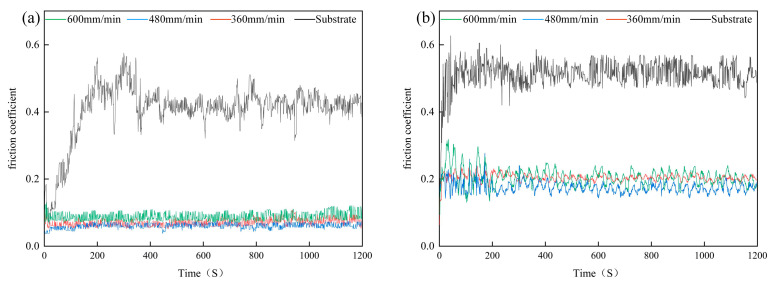
The friction coefficient of coatings and substrate at different temperatures: (**a**) room temperature, (**b**) 823 K.

The friction coefficient serves as a crucial indicator for selecting tool steel, as a lower friction coefficient ensures the reduction in wear and maintains the precision of the manufactured components. Table 7 presents the average friction coefficients of coatings and the substrate at different temperatures. The friction coefficients of the coatings were consistently lower than that of the substrate under different scanning speeds. The wear test possesses two stages: the running-in period and the steady-state period. The first period is the initial contact stage of the friction pair, requiring a greater force to slide, resulting in intense friction and significant fluctuations as shown in Figure 7. As wear continues, the contact points of the friction pair become smoother due to wear and plastic deformation of the samples, leading to a noticeable decrease in the friction coefficient, thus entering the second stage. During the steady-state friction stage, the contact surfaces of the friction pair undergo multiple interactions, resulting in more stable friction and a relatively constant friction coefficient. Figure 7a demonstrates that at room temperature, all coatings achieved the steady-state friction stage more quickly compared to the substrate and exhibited steady friction coefficients without the pronounced fluctuations observed in the substrate. The lower friction coefficients of coatings are mainly due to the production of oxide layers from Cr, Ti, and Zr elements within the coatings during wear tests, which produce a self-lubricating effect. In the condition of indoor temperature, the coating at a scanning speed of 480 mm/min has the lowest friction coefficient (0.06), which is one-fifth of the FeCoCrNiMnAl [34] coating (0.3).

At 823 K, the coatings’ friction coefficients remained lower than the substrate. At 823 K, the friction coefficients exhibited periodic fluctuations because the formation of oxide films composed of Cr, Ti, and Zr elements on the surface of coatings resulted in lower friction coefficient values. Under elevated temperature conditions, the momentary contact temperature between the friction pair and the coatings increased, leading to wear of the oxide films and a consequent rise in friction coefficients. Additionally, the oxidation of coatings was accelerated under elevated temperatures, facilitating the formation of new oxide layers and further reducing the friction coefficients. Thus, the friction coefficients of the coatings underwent periodic changes due to the continuous cycle of wear and regeneration at elevated temperatures. The coating at a scanning speed of 480 mm/min exhibited the lowest friction coefficients, both at room temperature and at 823 K.

**Figure 8:**
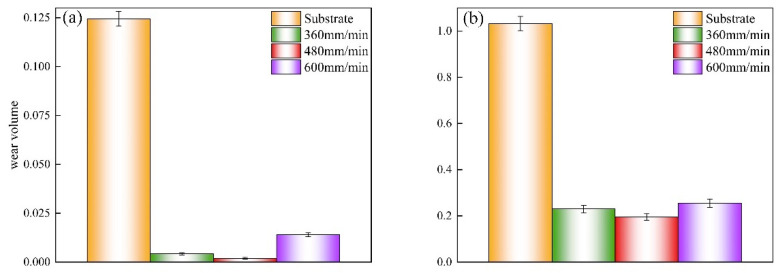
The wear volume of coatings and substrate: (**a**) room temperature, (**b**) 823 K.

Figure 8 reveals that under both room temperature and 823 K conditions, the wear volume of the coatings was substantially decreased compared to the substrate. Notably, the coating applied at a scanning speed of 480 mm/min reduced the wear volume by approximately 98% and 81% under room temperature and 823 K conditions, respectively. This significant enhancement is explained by the internal dislocation, fine grain, and solid solution strengthening within the coatings, contributing to their increased hardness. Additionally, the coatings’ inherent excellent self-lubricating properties also played a crucial role. However, at 823 K, this self-lubrication was compromised, leading to less ideal wear volume than under room temperature conditions, though the wear resistance still remained significantly superior to the substrate.

**Figure 9:**
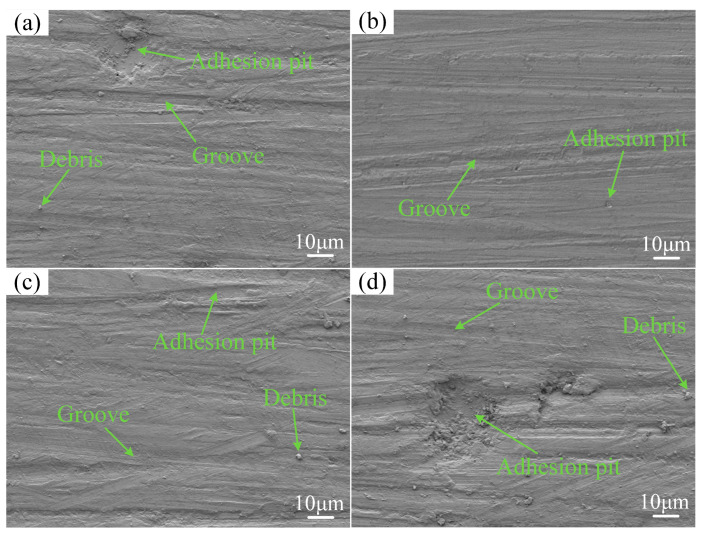
The wear morphology of coatings and substrate at room temperatures: (**a**) 360 mm/min; (**b**) 480 mm/min; (**c**) 600 mm/min; (**d**) substrate.

To gain insight into the wear mechanisms of the coatings and the substrate, the wear morphology was captured using SEM, as shown in Figure 9. Observations revealed that all coatings exhibited grooves and adhesion pits under room temperature wear tests, indicating that all coatings underwent abrasive wear and adhesive wear. Among them, the coating at a scanning speed of 480 mm/min had the shallowest grooves, and due to the small area of adhesion pits, no debris was observed. In contrast, the surface of the substrate exhibited deep and wide grooves. This phenomenon is explained by the fact that the substrate possesses lower hardness, which leads to plastic deformation under the pressure of the hard friction pair. Consequently, aggregation and welding occur, resulting in severe spalling on the worn surface due to relative motion. The surface morphology of the substrate shows significant plastic deformation, characterized by deep and wide fish-scale-like tears, accompanied by spalling pits and abundant wear debris. This indicates that the substrate experienced both abrasive wear and adhesive wear. Table 8 shows that both the coatings and substrate also underwent slight oxidative wear at room temperature.

**Figure 10:**
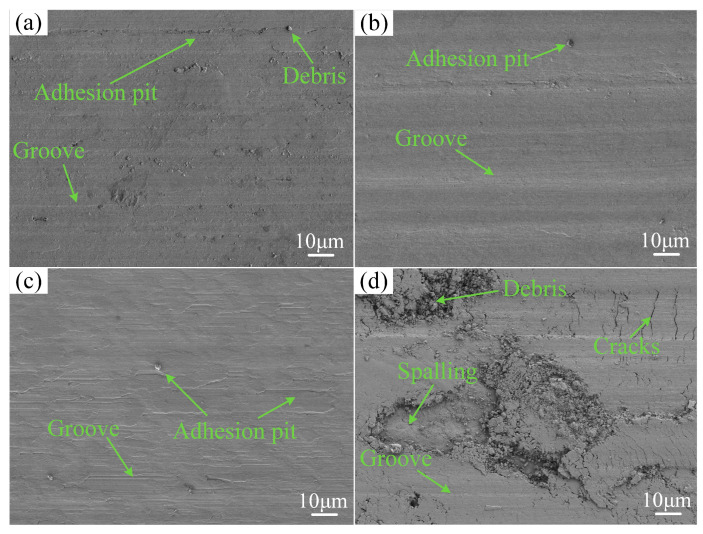
The wear morphology of coatings and substrate at 823 K: (**a**) 360 mm/min; (**b**) 480 mm/min; (**c**) 600 mm/min; (**d**) substrate.

Figure 10 shows that the coatings underwent both abrasive and adhesive wear at 823 K. However, as shown in Table 8, oxidative wear of the coatings increased under these conditions. Compared to indoor temperature, the coatings’ wear morphology was smoother, attributable to the continuous production of a lubricating oxide film during elevated temperature wear, which replaced the coating itself in contact with the friction pair. Figure 10d reveals that the substrate at 823 K exhibits significant debris, deeper grooves, cracks, and extensive spalling, indicating severe abrasive, adhesive, and fatigue wear. The oxygen content in the wear morphology of the substrate also increased significantly compared to room temperature, suggesting intensified oxidative wear. Regardless of the temperature, the coating at a scanning speed of 480 mm/min exhibited the smoothest wear morphology.

## 4. Conclusions

The FeCrNiTiZr HEA was fabricated on H13 steel via laser cladding. The microstructure and elevated temperature wear mechanism of the coatings were investigated. The findings are summarized as follows:

(1) The coatings consist of a BCC phase, with lattice distortion occurring in the phase structure, leading to a high dislocation density. The structure of the bonding zone is planar crystal, while the coating’s microstructure comprises dendrite crystals and cellular crystals.
(2) Through the combined effects of dislocation, fine-grain, and solid solution strengthening, the microhardness of the coatings at various scanning speeds is 3.5 to 4.2 times greater than that of the substrate.
(3) The coatings possess lower average friction coefficients than that of the substrate at different temperatures, and accordingly, the wear volume of the coatings is also lower. Compared to the substrate, the coatings undergo slight abrasive wear, adhesive wear, and oxidative wear at both room temperature and 823 K. In contrast, the substrate undergoes severe abrasive wear, adhesive wear, oxidative wear, and even fatigue wear.
(4) At a scanning speed of 480 mm/min, the coating exhibits the best performance, with the highest dislocation density compared to other coatings. The coating has the finest grain and the highest average microhardness (842 HV_0.2_), which is 4.2 times that of the substrate (200 HV_0.2_). The coating also has the lowest friction coefficient and the smallest wear volume, which is approximately 640% less than that of the substrate.

## Figures and Tables

**Table 1:** Chemical composition of H13 steel.

Element	C	Si	Mn	Cr	Mo	V	S	P	Fe
wt.%	0.32–0.45	0.80–1.20	0.20–0.50	4.75–5.50	1.10–1.75	0.80–1.20	≤0.03	≤0.03	Bal.

**Table 2:** Element contents of FeCrNiTiZr powder.

High-Entropy Alloy	Fe	Cr	Ni	Ti	Zr
wt.%	18.27	17.01	19.21	15.66	29.85

**Table 3:** The process parameters of laser cladding.

Laser Power	Scanning Speed	Overlap Rate	Spot Diameter	Shielding Gas	Shielding Gas Flow
1400 W	360 mm/min 480 mm/min 600 mm/min	30%	3 mm	argon	5 L/h

**Table 4:** *a*, *ε*, *ξ*, and *ρ* of Fe-Cr phases in coatings.

Coating	*a* (Å)	*ε*	*ξ*	*ρ* (cm^−2^)
360 mm/min	2.887	3.825 × 10^−3^	2.2511 × 10^−3^	2.30 × 10^11^
480 mm/min	2.890	4.868 × 10^−3^	2.4722 × 10^−3^	2.35 × 10^11^
600 mm/min	2.8835	2.6078 × 10^−3^	1.5126 × 10^−3^	1.50 × 10^11^

**Table 5:** β and θ of Fe-Cr phases in coatings.

Coating	*θ*(°)	*β*
360 mm/min	44.338	0.432
	64.477	0.32
	81.487	0.351
	98.089	0.283
480 mm/min	44.293	0.239
	64.221	0.304
	81.839	0.145
	97.698	0.271
600 mm/min	44.393	0.335
	64.204	0.288
	81.716	0.463
	97.959	0.202

**Table 6:** The chemical composition of coatings in grain boundary and grain (wt.%).

Points	Fe	Cr	Ni	Ti	Zr
1	54.3	11.4	12.3	5.8	16.2
2	62.9	15.4	9.5	3.3	8.9
3	53.6	11.7	11.6	5.1	18.0
4	63.8	16.4	8.0	3.3	8.5
5	54.6	10.5	12.1	5.8	17.0
6	65.5	15.2	7.7	3.2	8.4

**Table 7:** The average friction coefficient of coatings and substrate at different temperatures.

Temperature	Substrate	360 mm/min	480 mm/min	600 mm/min
Room temperature	0.42	0.07	0.06	0.09
823 K	0.52	0.20	0.17	0.21

**Table 8:** The chemical composition of wear morphology at different temperatures (wt.%).

Alloy	Temperatures	Fe	Cr	Ni	Ti	Zr	O	C	Si	V
360 mm/min	room temperature	35.71	15.47	17.35	8.52	19.05	3.90	-	-	-
	823 K	44.20	11.31	10.34	5.44	13.04	15.67	-	-	-
480 mm/min	room temperature	38.79	15.89	16.77	8.06	17.62	3.87	-	-	-
	823 K	41.53	11.39	11.34	6.29	15.06	14.39	-	-	-
600 mm/min	room temperature	35.62	15.23	16.85	8.27	19.12	4.91	-	-	-
	823 K	41.29	11.03	11.57	6.28	15.58	14.25	-	-	-
Substrate	room temperature	69.13	3.40	-	-	-	8.00	13.36	0.49	0.62
	823 K	69.59	3.98	-	-	-	21.39	3.75	0.75	0.84

## Data Availability

The original contributions presented in the study are included in the article, further inquiries can be directed to the corresponding author.
